# Implementing a multisector public-private partnership to improve urban hypertension management in low-and middle- income countries

**DOI:** 10.1186/s12889-022-14833-y

**Published:** 2022-12-19

**Authors:** Johannes Boch, Lakshmi Venkitachalam, Adela Santana, Olivia Jones, Theresa Reiker, Sarah Des Rosiers, Jason T. Shellaby, Jasmina Saric, Peter Steinmann, Jose M. E. Ferrer, Louise Morgan, Asha Barshilia, Edmir Peralta Rollemberg Albuquerque, Alvaro Avezum, Joseph Barboza, Yara C. Baxter, Luiz Bortolotto, Enkhtuya Byambasuren, Márcia Cerqueira, Naranjargal Dashdorj, Karina Mauro Dib, Babacar Guèye, Karim Seck, Mariana Silveira, Suely Miya Shiraishi Rollemberg, Renato W. de Oliveira, Tumurbaatar Luvsansambuu, Ann Aerts

**Affiliations:** 1 Novartis Foundation, Basel, Switzerland; 2 American Heart Association, Dallas, Texas USA; 3 Swiss Tropical and Public Health Institute, Basel, Switzerland; 4 University of Basel, Basel, Switzerland; 5 Secretaria Municipal da Saúde, São Paulo, Brazil; 6 Sociedade de Cardiologia do Estado de São Paulo & Hospital Alemão Oswaldo Cruz, São Paulo, Brazil; 7 Intrahealth, Dakar, Senegal; 8 YC Baxter, São Paulo, Brazil; 9 Sociedade Brasileira de Hipertensão, São Paulo, Brazil; 10 Mongolian Public Health Professionals’ Association, Ulaanbaatar, Mongolia; 11 Onom Foundation, Ulaanbaatar, Mongolia; 12 Ministère de la Santé et de l’Action Sociale, Dakar, Senegal; 13 Instituto Tellus, São Paulo, Brazil; 14 Iqvia, São Paulo, Brazil; 15 Capital City Health Department, Ulaanbaatar, Mongolia

**Keywords:** Population health, Primary care, Urban health, Public private partnership, Cardiovascular risk, Hypertension

## Abstract

**Background:**

Cardiovascular disease presents an increasing health burden to low- and middle-income countries. Although ample therapeutic options and care improvement frameworks exist to address its prime risk factor, hypertension, blood pressure control rates remain poor. We describe the results of an effectiveness study of a multisector urban population health initiative that targets hypertension in a real-world implementation setting in cities across three continents. The initiative followed the “CARDIO4Cities” approach (quality of Care, early Access, policy Reform, Data and digital technology, Intersectoral collaboration, and local Ownership).

**Method:**

The approach was applied in Ulaanbaatar in Mongolia, Dakar in Senegal, and São Paulo in Brazil. In each city, a portfolio of evidence-based practices was implemented, tailored to local priorities and available data. Outcomes were measured by extracting hypertension diagnosis, treatment and control rates from primary health records. Data from 18,997 patients with hypertension in primary health facilities were analyzed.

**Results:**

Over one to two years of implementation, blood pressure control rates among enrolled patients receiving medication tripled in São Paulo (from 12·3% to 31·2%) and Dakar (from 6·7% to 19·4%) and increased six-fold in Ulaanbaatar (from 3·1% to 19·7%).

**Conclusions:**

This study provides first evidence that a multisectoral population health approach to implement known best-practices, supported by data and digital technologies, and relying on local buy-in and ownership, can improve hypertension control in high-burden urban primary care settings in low-and middle-income countries.

**Supplementary Information:**

The online version contains supplementary material available at 10.1186/s12889-022-14833-y.

## Background

Hypertension or high blood pressure (BP) is a prime risk factor for cardiovascular disease (CVD) and is responsible for 10 million deaths per year [1]. An estimated 1·13 to 1·40 billion people suffer from high BP, a majority (66–75%) living in low- and middle-income countries (LMICs) [2]. Although a broad range of therapeutic options [3, 4] and quality of care improvement frameworks for hypertension are available, [5–7] BP control rates remain poor globally [2, 8].

Over the last two decades, LMICs have reported increased prevalence of hypertension due to changes in behavioral risk factors, diets and the initiating impact of demographic shifts and aging populations, ultimately contributing to an elevated burden of CVD [2]. On average, people in LMICs experience CVD at a younger age and with worse outcomes than in high-income countries [9]. In 2019, only 10% of hypertensive patients in LMICs achieved BP control [10], compared to on average 30–40% and up to 60% in high-income countries [11]. Low health literacy and limited access to BP screening and diagnosis, treatment, and follow-up hinder patients’ abilities to achieve BP control [2, 9, 12, 13]. As health systems in LMICs are mainly geared towards acute conditions, they are often insufficiently prepared to address the needs of patients with chronic diseases such as hypertension [12, 13]. Additionally, rapid and unplanned urbanization in LMICs yields large proportions of urban populations with poor access to health services and healthy lifestyle options [14].

**Fig. 1:**
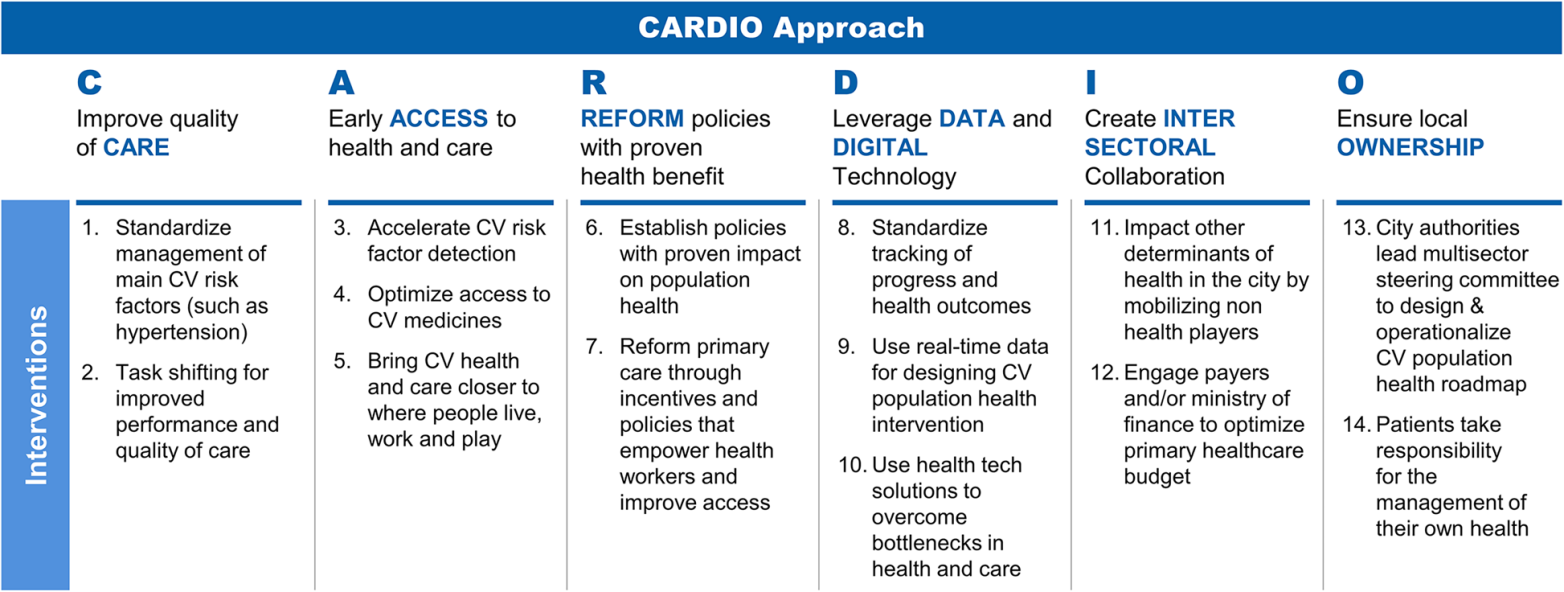
The six strategic pillars of the CARDIO4Cities approach

To address both health system weaknesses and the underlying determinants of hypertension, the health sector has to collaborate with sectors such as education, sports, food and agriculture, urban planning, technology and finance [15, 16]. Understanding that reengineering health and care delivery for hypertension is a complex and multilayered undertaking that requires efforts of more than one entity, we implemented a multisectoral urban health initiative to reduce the burden of hypertension. It brings together public and private entities with diverse expertise and resources in an approach owned by local authorities and shaped by local priorities and data. The aim was to build evidence that intersectoral collaboration is feasible, can improve health outcomes and contribute to cardiovascular (CV) population health in cities [16]. The approach, called CARDIO4Cities [17] was described before and applies a comprehensive strategy based on six pillars: quality of Care, early Access, policy Reform, Data and digital technology, Intersectoral collaboration, and local Ownership (Fig. 1).

Three cities - Dakar (Senegal), São Paulo (Brazil), and Ulaanbaatar (Mongolia) - were chosen to pioneer the CARDIO4Cities approach. The cities represent different urban contexts across three continents, and were selected based on their high hypertension and CVD burden, as well as the political will to address hypertension [17]. To evaluate impact, real-world data on the “cascade” of hypertension diagnosis, treatment and control was collected at primary healthcare level in the public health systems of the pioneer cities. Here, we report initial results of this effectiveness study of the CARDIO4Cities approach in a real-world implementation setting based on data collected from the initiative’s onset in 2018 through December 2019.

## Methods

### Setting up patient and public involvement

In each city, implementation of the CARDIO4Cities approach was co-designed between the Novartis Foundation and local authorities and partners. This included regular meetings of joint technical working groups to identify initial demonstration sites within the cities and conduct a situational analysis to explore unmet needs and gaps in response to the growing burden of hypertension [18]. Public representatives such as health managers, doctors, nurses, community health workers, patients, public authorities and community representatives were closely involved throughout the health system and baseline assessment, the planning of interventions, and the feedback reviews on progress, results and optimization opportunities [18].

### Establishing data collection and baseline metrics

Due to the collaborative development and real-time embedment of activities in the existing health system, the initiative could not be implemented as a trial. Similarly, extensive baseline data was not available, as data collection prior to implementation was not standardized. Therefore, the program first established a process for data collection, based on which the program impact was continuously monitored, evaluated, and documented. In-country partners, with support and oversight from a global evaluation partner, were responsible for data collection, management, and analysis. In the absence of digital health records, data were collected by data clerks from hypertension registries in primary health facilities or from paper-based patient records of patients who visited the health centers during the urban population health initiative period, except for Ulaanbaatar, where records were also abstracted for patients diagnosed prior to the initiative regardless of whether they had a visit during the initiative’s period. The primary health facilities were called family health centers in Ulaanbaatar, health centers or health posts in Dakar, and primary health units (Unidade Básica de Saúde) in São Paulo (see S1 Table for details on coverage). To assess progress in hypertension management, data related to hypertension outcomes (diagnosis, treatment, and control) and, if available, CV risk status as defined by physicians on initial patient engagement, were collected in line with local guidelines (S2 Table). Patient identification, execution of BP measurements, and clinical definitions of hypertension and control followed local guidelines and healthcare structures. Consequently, BP was commonly measured by health professionals and hypertension diagnosis was defined as BP ≥ 140 and/or 90 mmHg in Dakar and São Paulo and as BP ≥ 130 and/or 80 mmHg in Ulaanbaatar (S2 Table). In the case of Ulaanbaatar, the Mongolian Ministry of Health had adapted the threshold of the definition for hypertension (Order No. A/286) to the recommendations of the American Heart Association from 2017 [19, 20]. Definitions of the initiative’s metrics are provided in Tabs S2 and S3. When data collection could not cover all primary health facilities in a city, a sample of facilities was selected based on guidance from local partners and authorities (S2 Table). In the case of Ulaanbaatar, data was collected from a random sample of 23 family health centers out of the city’s 142 such facilities. In São Paulo, patient medical records were analyzed in six of the 45 primary care units in the participating district. Those were selected following guidance from the city Secretary of Health, to represent the multiple primary care delivery models in São Paulo. In Dakar eventually all 66 health centers and health posts were included in data collection.

The initiative was launched at different times in the different cities, following variable speeds of concluding agreements with local authorities and partners, finalizing needs assessments, and establishing independent oversight of the outcome measurement. Therefore, results were available at different time points, with Ulaanbaatar reporting data as of the first quarter (Q1), Dakar the second quarter (Q2) and São Paulo the fourth quarter (Q4) of year 2018. The data collected in these quarters were defined as baseline for this evaluation.

### Implementing the CARDIO4Cities approach

With relevant stakeholders and partners, such as representatives from primary care, district authorities, local professional associations and implementation partners, city authorities established a tailored intervention package based on the CARDIO4Cities approach. Interventions were designed to implement global best-practices for hypertension and CVD management, such as the World Health Organization (WHO) HEARTS package, [21] the M.A.P. framework™, [6] and the World Heart Federation roadmap to reduce high BP [22],as well as local guidance, policies, guidelines and data [23]. Intervention strategies varied and included: standardizing hypertension diagnosis and management at primary health level through clinical decision support systems, trainings on medical aspects, risk factors and lifestyle change, and online continuous medical education for providers, including health workers in clinics, community health workers, and pharmacists; engaging non-traditional health players such as football and samba clubs, schools, and workplaces [24] to maximize opportunities for hypertension detection and increase CV risk health literacy; and, increasing opportunities for physical exercise or healthy food options in the city [17]. Where in Brazil free treatment was available to all according to local policy, in other cities the partnership worked with local authorities to include hypertension medicines into essential drug lists. No medication was provided by the initiative. No medication was provided by the initiative. The core interventions in each city are summarized in supplement materials (S3 Table). Implementation was continuously refined based on ongoing data evaluation with the local partners. While initially activities covered limited city areas or districts, coverage was gradually increased to include the entire cities of Dakar and Ulaanbaatar, and two districts of São Paulo (reaching 1·3, 1·2, and 1·0 million people, respectively) within 19, 20, and 15 months, respectively (S1 Table).

### Outcome evaluation

In this manuscript, we describe the changes in rates across the hypertension care cascade throughout the initial implementation phase of CARDIO4Cities, up to Q4 2019. We thus conclude the initial reporting period before onset of the Covid-19 pandemic. Data on the hypertension care cascade were collected in quarterly, cross-sectional analyses of patient files and systolic BP was collected longitudinally from the same source. Deidentified patient data were extracted from health records of adults (aged > 18 years) with a diagnosis of hypertension, who had minimum one visit at the facility during the reporting period. Data were summarized as means and 95% confidence intervals (CI) calculated for continuous data (e.g., net change in systolic BP), and as counts and percentages for categorical variables (e.g., sex). Where this information was available in the medical records, health outcomes were stratified by age, sex, BP category and baseline CV risk. The association between outcomes and the missingness of patient characteristics were examined using Pearson’s chi-square test. For cell counts less than five, Fischer’s exact test was used. Given the primary emphasis on improving routine surveillance of hypertension cascade indicators, data collection was limited to minimally essential patient characteristics, precluding comprehensive assessment of predictors. Crude odds ratios with Fisher 95% CI were estimated from aggregate data to reflect sizes for outcomes (receiving treatment, achieving BP control) related to the four key baseline patient characteristics. Cumulative data on health outcomes were reported by quarter during the reporting period. Statistical trend testing was not conducted on metrics with fewer than 10 data points due to insufficient power to detect reasonable-sized effects [25]. Net change in average systolic BP was calculated as the difference in the BP values between first and last patient visits and summarized as mean change with 95% CI. Unless stated otherwise, an alpha level of 0·05 was used to assess statistical significance.

### Software

Data were analyzed using Microsoft® Excel® for Microsoft 365, Microsoft, Redmond, WA, USA, Stata/SE 15·1 for Windows, StataCorp LLC, College Station, TX, USA, and R-4·1·0. Odds ratios were calculated using the epitools package in R [26].

### Research ethics approval and patient consent

Approvals were obtained from government entities and/or local ethics committees. All data collection and processing has been carried out in accordance with local regulations and relevant guidelines. Local protocols have been relevant authorities or ethic committees. The ethics approvals for the respective countries are: Letter No. 1/158 dated February 21st, 2018 from the Capital City department of health for Ulaanbaatar, SEN 18/79 and SEN 19/14 from the Comité National d’Ethique pour la Recherche en Santé for Dakar, and Comitê de Ética – Secretaria Municipal da Saúde, São Paulo (CEP-SMS); 3·818·858 for São Paulo with the latter requiring informed consent. In Dakar and Ulaanbaatar, informed consent was not required. In Ulaanbaatar, monitoring and evaluation of data reported in this paper was considered evaluation, and not humans subjects research, approval for data collection and waiver of informed consent was granted by the Ulaanbaatar health department. In Dakar, informed consent was waived by the Ministere de la Santé e de l’Action Social du Senegal. The initiative supported the establishment of a hypertension registry. The data presented in this paper was extracted from this registry. Data was only shared as aggregated and anonymized work product.

### Data availability statement

The global indicator framework is provided in S2 supplementary File. The sharing of individual-level data is not possible due to the restriction of local approvals and ethical committees. Only the local measurement and evaluation partners were granted approval to store and analyze individual-level data. Therefore, data was stored and analyzed locally for all cities. Only aggregated and de-identified data was shared with global partners. Data was collected from standard procedures in the primary healthcare system and is thus under the jurisdiction of the respective health authorities. Data was collected by the Mongolian Public Health Professionals’ Association and owned by and available exclusively from the Capital City Department of Health for Ulaanbaatar. In Dakar, data is owned by the Division de la Statistique et de l’Information Sanitaire (DSIS), part of the Direction de la Planification de la Recherche et de la Statistique (DPRS) within the Ministère de la Santé et de l’Action Sociale. Data is stored on their behalf by Intrahealth Dakar. For São Paulo, data is stored by IQVIA Brasil Ltda., on behalf of the data owner, Secretaria Municipal da Saúde. Access to data for all cities needs to be requested from the respective government departments. Support can be provided by the local implementing partners (Intrahealth for Dakar, Instituto Tellus for Brazil, and Onom Foundation for Ulaanbaatar) and the Novartis Foundation.

## Results

At the start of the initiative, each city worked with its professional societies and experts to simplify care algorithms for hypertension management and provided decision support tools to primary health providers. In the reported timeframe (2018 until December 2019), information on simplified care algorithms for hypertension management was distributed to a total of 232 participating health facilities, covering 100% in Ulaanbaatar (142) and Dakar (66) and 5% in São Paulo (24 out of 468). Training was provided in each city, covering healthcare, operational and managerial practices for 301 management staff (43 health center managers and 258 district health managers), 2516 health providers (1018 physicians and 1498 nurses), and 839 community health workers. Additionally, 580 pharmacists were trained in Ulaanbaatar and São Paulo. Overall, 3546 community events were organized, reaching approximately 88,000 individuals (S5 Table).

### Ulaanbaatar

Between Q1 2018 and Q3 2019, 11,189 patients diagnosed with hypertension were enrolled in 23 family health centers, of whom 584 (5·2%) were newly diagnosed during the reporting period. Most patients were 45–69 years old (65·3%), female (62·8%), and had low or moderate/intermediate baseline CV risk (58·2%) (Table 1).

**Table 1:** Profile of patients at their first visit during reporting period Q1 2018 – Q3 2019, Ulaanbaatar

	Diagnosed		Treated		Controlled BP (at the last visit)		Among all patients, with data	
	Total ***N*** = 11,189	%	Total ***N*** = 10,075	%	Total ***N*** = 1986	%	Treated Crude OR (95% CI)	BP Controlled Crude OR (95% CI)
Age (years)								
18–29	150	1·3	83	0 8	8	0·4	REF	REF
30–44	1050	9·4	800	7·9	107	5·4	2·6 (1·8, 3·7) ^¥¥¥^	2·0 (0·9, 4·9)
45–59	3811	34·1	3385	33·6	579	29·2	6·4 (4·5, 9·1) ^¥¥¥^	3·2 (1·6, 7·5) ^¥¥¥^
60–69	3496	31·2	3279	32·5	639	32·3	12·2 (8·4, 17·5) ^¥¥¥^	4·0 (1·9, 9·4) ^¥¥¥^
70–79	1918	17·1	1800	17·9	463	23·4	12·3 (8·3, 18·1) ^¥¥¥^	5·6 (2·8, 13·4) ^¥¥¥^
≥ 80	729	6·5	693	6·9	190	9·6	15·5 (9·5, 25·5) ^¥¥¥^	6·2 (3·0, 15·0) ^¥¥¥^
Missing age information	35	0·3	35^*^	0·3	0**	0·0	NA	NA
Sex							REF: MEN	REF: MEN
Women	7029	62·8	6371	63·2	1344	67·8	1·2 (1·0, 1·4) ^¥¥^	1·3 (1·2, 1·5) ^¥¥¥^
Missing sex information	13	0·1	12	0·1	3	0·2	NA	NA
BP Categories (mmHg)								
< 120 and < 80 mmHg	362	3·2	335	3·3	90	4·5	REF	REF
120–129 or 80–84 mmHg	704	6·3	672	6·7	170	8·6	1·7 (1·0, 3·0) ^¥^	1·0 (0·7, 1·3)
130–139 or 85–89 mmHg	2252	20·1	1871	18·6	331	16·7	0·4 (0·3, 0·6) ^¥¥¥^	0·5 (0·4, 0·7) ^¥¥¥^
140–159 or 90–99 mmHg	3632	32·5	3184	31·6	433	21·9	0·6 (0·4, 0·9) ^¥¥^	0·4 (0·3, 0·5) ^¥¥¥^
160–179 or 100–109 mmHg	2185	19·5	2014	20·0	328	16·6	0·9 (0·6, 1·5)	0·5 (0·4, 0·7) ^¥¥¥^
≥ 180 or ≥ 110 mmHg	726	6·5	680	6·7	109	5·5	1·2 (0·7, 2·0)	0·5 (0·4, 0·7) ^¥¥¥^
Missing BP information	1328	11·9	1319***	13·1	525***	26·5	NA	NA
CV Risk^a^								
Low	3636	32·5	3511	34·8	690	34·8	REF	REF
Moderate/Intermediate	2874	25·7	2816	28·0	406	20·5	1·7 (1·3, 2·4) ^¥¥¥^	0·7 (0·6, 0·8) ^¥¥¥^
High	565	5·0	547	5·4	99	5·0	1·1 (0·7, 1·9)	0·9 (0·7, 1·1)
Missing	4144	37·0	3201***	31·8	791**	39·9	NA	NA

Chi-square test or statistical significance for missingness of patient characteristic data *p < 0·05, **p < 0·01, ***p < 0·001

*BP* blood pressure, *CI* confidence intervals, *CV* cardiovascular, *NA* not applicable, *OR* odds ratio, *REF* reference

^a^CV risk was defined according to city-specific guidelines (see S3 Table)

Crude odds ratios and 95% CIs estimated only among patients with non-missing data and rounded to one decimal place; ^¥^*p* < 0·05, ^¥¥^*p* < 0·01, ^¥¥¥^*p* < 0·001

**Fig. 2:**
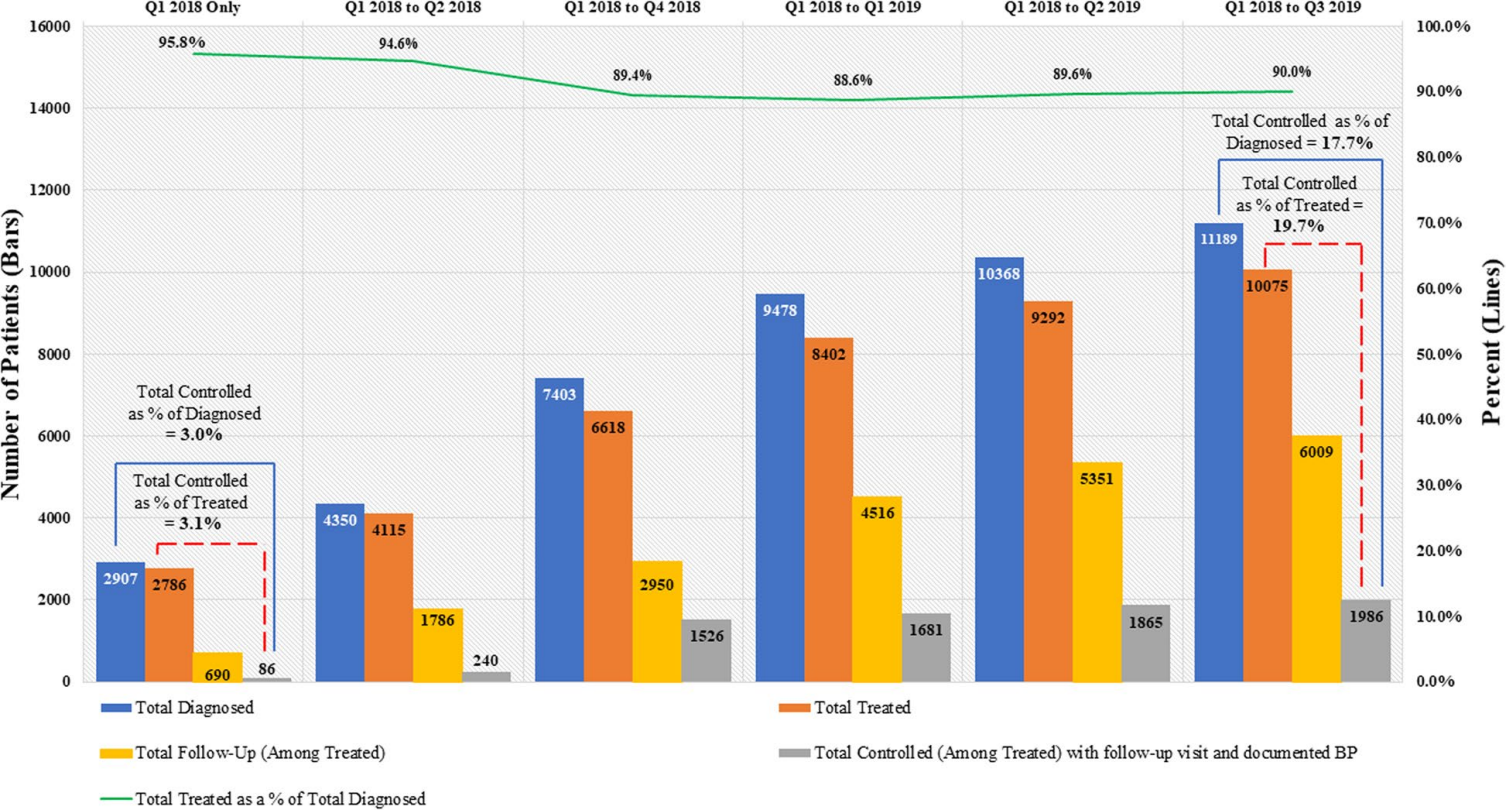
Cumulative coverage and progress in patients diagnosed, treated, and controlled for hypertension, Ulaanbaatar, Q1-Q2 2018, Nov 2018 - Q3 2019

Among patients diagnosed with hypertension, there was an increase in BP control rate from 3·0% at the start of the reporting period (Q1 2018) to 17.7% in Q3 2019. While 2786 (95·8%) of the documented hypertensive patients were treated with medication in Q1 2018, only 86 (3·1%) of those patients had their BP controlled at the last visit in that quarter. At the end of the reporting period in Q3 2019, 10,075 (90·0%) of the 11,189 documented hypertensive patients were treated with antihypertensive medications and 1986 of them achieved BP control at the last visit, reflecting an increase from 3·1% to 19·7% during the reporting period (Fig. 2). Follow-up rates amongst treated patients (at least two visits during the reporting period) increased from 24·8 to 59.6% over the same period. Table 1 summarizes the characteristics of all hypertensive patients reported between Q1 2018 and Q3 2019, at the time of their first visit.

Most patients with controlled BP at their last known visit, were aged 45–69 (61·5%) years at baseline and female (67·8%). Only a quarter (25·5%) had moderate or high baseline CV risk (Table 1). The odds of achieving BP control increased with age and women presented 30% greater odds of achieving control than men (OR = 1·3, 95% CI: 1·2–1·5, *p* < 0.001). The odds of achieving BP control were significantly lower for patients with baseline BP ≥130/≥85 (OR = 0.4–0.5, *p* > 0.001 for all categories), and for those with a moderate baseline CV risk (OR = 0·7, 95% CI: 0·6–0·8, *p* < 0.001) (Table 1). On average, mean systolic BP was reduced by 4·65 mmHg (95% CI: 4·18–5·12) in 9586 hypertensive patients who had at least two documented values during the reporting period.

### Dakar

The total number of patients diagnosed with hypertension increased from Q2 2018 to Q4 2019. Initially, data from only 470 hypertensive patients was captured. By Q4 2019, that number increased to 6056 patients across 66 primary health centers. Of these, 5215 (86·1%) were newly diagnosed, and the majority were 45–69 years old (62·6%) and female (77·1%). Most patients (66·4%) had a moderate or high CV risk at baseline (Table 2).

**Table 2:** Profile of patients at their first clinic visit during reporting period Q2 2018 – Q4 2019, Dakar

	Diagnosed		Treated		Controlled BP (at the last visit)		Among all patients with data	
	Total ***N*** = 6056	%	Total ***N*** = 5236	%	Total ***N*** = 1016	%	Treated Crude OR (95% CI)	BP controlled Crude OR (95% CI)
Age (years)								
18–29	110	1·8	84	1·6	9	0·9	REF	REF
30–44	797	13·2	652	12·5	115	11·3	1·4 (0·8, 2·3)	1·9 (0·9, 4·4)
45–59	2017	33·3	1753	33·5	333	32·8	2·1 (1·2, 3·3) ^¥¥^	2·2 (1·1, 5·0) ^¥^
60–69	1777	29·3	1584	30·3	334	32·9	2·5 (1·5, 4·1) ^¥¥¥^	2·6 (1·3, 5·9) ^¥¥^
70–79	840	13·9	743	14·2	159	15·6	2·4 (1·4, 3·9) ^¥¥¥^	2·6 (1·3, 6·0) ^¥¥^
≥ 80	227	3·7	184	3·5	32	3·1	1·3 (0·7, 2·4)	1·8 (0·8, 4·6)
Missing age information	288	4·8	236*	4·5	34*	3·3	NA	NA
Sex							REF: MEN	REF: MEN
Women	4669	77·1	4071	77·8	806	79·3	1·3 (1·1, 1·5) ^¥¥^	1·2 (0·9, 1·4)
Missing sex information	0	0·0	0	0·0	0	0·0	NA	NA
BP Categories (mmHg)								
< 120 and < 80 mmHg	146	2·4	132	2·5	41	4·0	REF	REF
120–129 or 80–84 mmHg	313	5·2	280	5·3	85	8·4	0·9 (0·4, 1·8)	1·0 (0·6, 1·5)
130–139 or 85–89 mmHg	656	10·8	563	10·8	152	15·0	0·6 (0·3, 1·2)	0·8 (0·5, 1·2)
140–159 or 90–99 mmHg	2207	36·4	1923	36·7	427	42·0	0·7 (0·4, 1·3)	0·6 (0·4, 0·9) ^¥^
160–179 or 100–109 mmHg	1527	25·2	1324	25·3	218	21·5	0·7 (0·4, 1·2)	0·4 (0·3, 0·6) ^¥¥¥^
≥ 180 or ≥ 110 mmHg	1112	18·4	986	18·8	90	8·9	0·8 (0·4, 1·5)	0·2 (0·1, 0·4) ^¥¥¥^
Missing BP information	95	1·6	28***	0·5	3***	0·3	NA	NA
CV Risk^a^								
Low	1119	18·5	1007	19·2	237	23·3	REF	REF
Moderate/Intermediate	1564	25·8	1436	27·4	282	27·8	1·2 (0·9, 1·6)	0·8 (0·7, 1·0) ^¥^
High	2459	40·6	2295	43·8	385	37·9	1·6 (1·2, 2·0) ^¥¥¥^	0·7 (0·6, 0·8) ^¥¥¥^
Missing	914	15·1	498***	9·5	112***	11·0	NA	NA

Chi-square test for statistical significance for missingness of patient characteristic data *p < 0·05, **p < 0·01, ***p < 0·001

*BP* blood pressure, *CI* confidence intervals, *CV* cardiovascular, *NA* not applicable, *OR* odds ratio, *REF* reference

^a^CV risk was defined according to city-specific guidelines (see S3)

Crude odds ratios and 95% CIs estimated only among patients with non-missing data and rounded to one decimal place; ^¥^*p* < 0·05, ^¥¥^*p* < 0·01, ^¥¥¥^*p* < 0·001

**Fig. 3:**
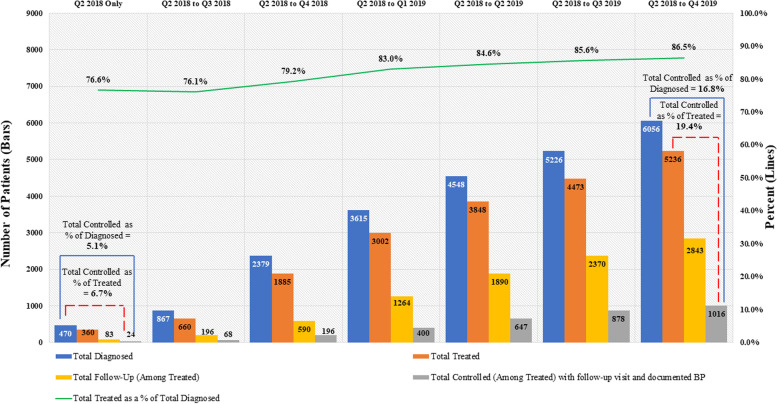
Cumulative coverage and progress in patients diagnosed, treated, and controlled for hypertension, Dakar, Q2 2018 - Q4 2019

Among patients diagnosed with hypertension, BP control rates increased from 5·1% at the start of the initiative (Q2 2018) to 16·8% in Q4 2019. In Q2 2018, 360 (76·6%) hypertensive patients were treated with medication; however, only 24 of them achieved BP control at the last visit. At the end of the reporting period (Q4 2019), 5236 (86·5%) of the documented 6056 hypertensive patients were treated with antihypertensive medications and 1016 of them achieved BP control at the last visit, reflecting an increase in BP control rate from 6·7% to 19·4% during the initiative (Fig. 3). Follow-up rates amongst treated patients increased from 23·1% to 54·3% over the same period. Table 2 summarizes the characteristics of all hypertensive patients recorded between Q2 2018 and Q4 2019, at the time of their first visit.

The majority of patients who achieved BP control at their last visit, were aged 45–69 (65·6%) years, were female (79·3%), and had moderate or high baseline CV risk (65·6%). Significant differences in BP control were observed by age (OR = 2·2–2·6 for age groups ≥45 and < 80 years old), baseline CV risk with decreased odds of control for moderate (OR = 0·8, 95% CI: 0·7–1·0, *p* < 0.05) or high risk (OR = 0·7, 95% CI: 0·6–0·8, *p* < 0.001), BP values of 140–159 or 90–99 mmHg (OR = 0·6, 95% CI: 0·4–0·9, *p* < 0.05), 160–179 or 100–109 mmHg (OR = 0·4, 95% CI: 0·3–0·6, *p* < 0.001) and ≥ 180 or ≥ 110 mmHg (OR = 0·2, 95% CI: 0·1–0·4, *p* < 0.001) (Table 2). On average, mean systolic BP was reduced by 15 mmHg (95% CI: 14·10–15·94 mmHg) among the 2893 patients who had at least two documented values during the reporting period.

### São Paulo

From Q4 2018 to Q4 2019, 1752 hypertensive patients were reported across the sample of six primary health centers in the Itaquera district. Of those, 209 (11·9%) were newly diagnosed during the study period. As in the other cities, the majority of patients were aged 45–69 (59·4%) years and female (70·3%), while the baseline CV risk assessment was missing for most (87·0%) of the patients (Table 3).

**Table 3:** Profile of patients at their first clinic visit during reporting period Q4 2018 – Q4 2019, São Paulo

	Diagnosed		Treated		Controlled (at the last visit)		Among all patients with data	
	Total ***N*** = 1752	%	Total ***N*** = 1461	%	Total ***N*** = 456	%	Treated Crude OR (95% CI)	BP controlled Crude OR (95% CI)
Age (years)								
18–29	13	0·7	7	0·5	3	0·7	REF	REF
30–44	152	8·7	111	7·6	34	7·5	2·3 (0·6, 8·6)	1·0, (0·2,5·7)
45–59	488	27·9	423	29·0	138	30·3	5·5 (1·5, 20·0) ^¥¥¥^	1·3 (0·3, 7·5)
60–69	552	31·5	461	31·6	135	29·6	4·3 (1·2, 15·4) ^¥¥^	1·1 (0·3, 6·2)
70–79	417	23·8	350	24·0	112	24·6	4·5 (1·2, 16·0) ^¥¥^	1·2 (0·3, 7·0)
≥ 80	130	7·4	109	7·5	34	7·5	4·4 (1·1, 17·0) ^¥¥^	1·2 (0·3, 7·1)
Missing age information	0	0·0	0	0·0	0	0·0	NA	NA
Sex							REF: MEN	REF: MEN
Women	1232	70·3	1045	71·5	316	69·3	1·3 (1·1, 1·8) ^¥^	0·9 (0·7, 1·2)
Missing sex information	44	2·6	32*	2·2	10	2·2	NA	NA
BP Categories (mmHg)								
< 120 and < 80 mmHg	141	8·0	113	7·7	43	9·4	REF	REF
120–129 or 80–84 mmHg	326	18·6	261	17·9	110	24·1	1·0 (0·6, 1·7)	1·2 (0·7, 1·8)
130–139 or 85–89 mmHg	212	12·1	175	12·0	59	12·9	1·2 (0·7, 2·1)	0·9 (0·5, 1·4)
140–159 or 90–99 mmHg	500	28·5	415	28·4	125	27·4	1·2 (0·7, 2·0)	0·8 (0·5, 1·2)
160–179 or 100–109 mmHg	365	20·8	330	22·6	93	20·4	2·3 (1·3, 4·1) ^¥¥^	0·8 (0·5, 1·2)
≥ 180 or ≥ 110 mmHg	121	6·9	113	7·7	26	5·7	3·5 (1·5, 9·2) ^¥¥^	0·6 (0·3, 1·1)
Missing BP information	87	5·0	54***	3·7	0***	0·0	NA	NA
CV Risk^a^								
Low	34	1·9	32	2·2	18	3·9	REF	REF
Moderate/Intermediate	45	2·6	41	2·8	19	4·2	0·6 (0·1, 4·8)	0·7 (0·2, 1·7)
High	149	8·5	130	8·9	47	10·3	0·4 (0·0, 1·9)	0·4 (0·2, 0·9) ^¥^
Missing	1524	87·0	1258*	86·1	372***	81·6	NA	NA

Chi-square test for statistical significance for missingness of patient characteristic data *p < 0·05, **p < 0·01, ***p < 0·001

*BP* blood pressure, *CI* confidence intervals, *CV* cardiovascular, *NA* not applicable, *OR* odds ratio, *REF* reference

^a^CV risk was defined according to city-specific guidelines (see S3)

Odds ratios and 95% CIs estimated only among patients with non-missing data and rounded to one decimal place; ^¥^*p* < 0·05, ^¥¥^*p* < 0·01, ^¥¥¥^*p* < 0·001

**Fig. 4:**
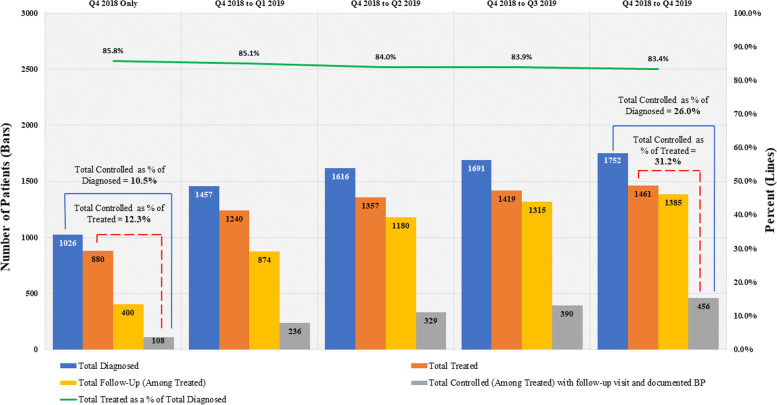
Cumulative coverage and progress in patients diagnosed, treated, and controlled for hypertension, São Paulo, Q4 2018 – Q4 2019

Among patients diagnosed with hypertension, BP control rates increased from 10·5% at the start of the initiative to 26·0% in Q4 2019. At the start, 880 (85·8%) of the 1026 patients with documented hypertension were treated with medication, and 108 (12 3%) achieved BP control. Treatment rates remained stable throughout the implementation period, ranging between 83·4% and 85·8%, and follow-up rates increased from 45·5% in Q3 2018 to 94·8% in Q4 2019. At the end of the reporting period, 456 of 1461 patients treated with medication achieved BP control at their last visit, representing an increase in BP control from 12·3% to 31·2% in a little more than one year of implementation (Fig. 4).

Table 3 summarizes the characteristics of hypertensive patients recorded between Q4 2018 and Q4 2019 (*n* = 1752). The majority of patients with controlled BP were 45–69 years old (59·9%) and female (69·3%). While data for baseline CV risk was missing for 81·6% of patients, 14·5% of those with available data had moderate or high CV risk. Patients with high baseline CV risk had 60% lower odds (95% CI: 0·2–0·9, *p* < 0.05) of achieving BP control than those with low CV risk. On average, mean systolic BP was reduced by 3·05 mmHg (95% CI: 1·72–4·38) in 1073 hypertensive patients with at least two documented measurements during the reporting period.

## Discussion and conclusion

The multidisciplinary public private partnership established between the Novartis Foundation and local authorities in Ulaanbaatar, Dakar, and São Paulo aimed to reengineer the way hypertension and its underlying determinants are addressed in urban populations, by translating widely available evidence into real-world practice. As CARDIO4Cities aimed at long term sustainability, we paid special attention to tailoring the initiative to local contexts, address jointly identified needs, and position local authorities to lead the initiative from the start. The results of this study in a real-world implementation setting provide first evidence that a comprehensive, data-driven approach such as CARDIO4Cities, targeting early access and quality of care, and using data to guide decision-making, can rapidly improve health outcomes. BP control rates in patients treated with antihypertensive medications almost tripled in São Paulo (from 12·3% to 31·2%) and Dakar (from 6·7% to 19·4%) and increased six-fold in Ulaanbaatar (from 3·1% to 19·7%) after relatively short periods of implementation. Based on existing evidence [6, 22], we believe that the initiative’s interventions contributed to improved BP control rates by strengthening performance of both health systems (through optimized and accelerated hypertension diagnosis, standardized hypertension management, as well as regular monitoring of progress and outcomes) and health professionals (through e.g. continuous medical education, clinical decision support systems and task-sharing with pharmacists and community health workers). But also, the increased engagement of patients in the management of their own health must have contributed. Women were overrepresented in all patient populations (from 62.8% in Ulaanbaatar to 77.1% in Dakar). As our data were extracted from medical records, this indicates that across all three cities, women were more likely to visit primary care points. Similarly, they were more likely to receive treatment and in Ulaanbaatar and Dakar, more likely to achieve control. We hypothesize that men may face greater stigma associated with seeking care or medical help, or that the overrepresentation of men in the workforce and long working days may prevent them from accessing care. To address this gender-gap in care seeking, our initiative specifically engaged football clubs in São Paulo. Notably, São Paulo is the only city included in our initiative, where the odds for men receiving hypertensive treatment to control their BP did not differ from those of women. Future population health initiatives should investigate how men can be better reached, to ensure that the entire population can benefit from the interventions.

In Ulaanbaatar, improvements in hypertension management (accelerated detection, standardization of care, continuous medical education, and clinical decision support systems for health workers), coupled to budget increases for primary health centers, allowed diagnosis, treatment, and long-term follow-up for hypertension to improve. Despite an average reduction of 4·65 mmHg in systolic BP, control rates in Ulaanbaatar only reached 19·7%. Based on conversations with our local partners, we hypothesize that in addition to the lower threshold for defining BP control in Mongolia (< 130/80 mmHg), irregular and insufficient supply of antihypertensive medications and high salt consumption may have been an important barrier to hypertension control. Following this assumption, our findings illustrate the importance of both improving access to medicines and quality of healthcare.

In Dakar, CARDIO4Cities improved hypertension detection and management through simplified standard treatment algorithms, and task shifting to community health workers and nurses. While previously, the health system could not estimate the number of people with hypertension, or monitor treatment or control rates, during our initiative health records were standardized and a first hypertension registry was created that now includes data from over 6000 patients diagnosed in 15 months. We attribute this success to optimized BP measurement opportunities, within and outside the health system, during community outreach, and in the workplace [24]. As health services for patients with chronic conditions in Dakar were almost inexistent at the start of our initiative in Dakar it is not unexpected that 86·1% of the patients were newly diagnosed during this initiative, demonstrating the high importance to accelerate detection and couple it to prompt referral into care in such settings.

In São Paulo, following the country’s longstanding focus on primary care and family health (Saúde da Família), the public health system offers solid infrastructure, care and operational processes, as well as free medicines for all [27]. Yet, BP control rates were low at the start of the initiative. After only one year of CARDIO4Cities, control rates almost tripled. We believe that this results from the continuous efforts to improve quality of hypertension management, amongst others by standardizing guidelines and their translation into standardized algorithms of care [28, 29] and roll these out to the entire health corps of the city through online continued medical education, in person trainings, and clinical decision support systems [30]. Pharmacists were also engaged, offering patient counseling and adherence support, and best practices for patient management were discussed in multidisciplinary team meetings. Early diagnosis was improved by equipping every primary health center with a BP screening corner, offering BP measurement to all adults visiting or consulting the facility [30]. Engagement of local champions in São Paulo, such as football and samba clubs or lifestyle ambassadors, enhanced people’s awareness about CV risk, and accelerated diagnosis. Digital technology helped in connecting people screened at extramural sites such as metro stations, to the health system [31, 32]. The initiative also optimized health center performance with management tools including organization panels, target planning and automated data reports, to facilitate data-based decision-making.

Data on the hypertension care cascade were used to monitor interventions and inform decision-making. Population surveys were available across cities and were used to inform target setting [33–35]. These surveys were however insufficient to inform outcomes during implementation due to inconsistent methodology across countries (e.g. self-referred hypertension diagnosis versus reported BP values), and a lack of in-country consensus on their quality and usefulness for clinical practice. As a main objective of this initiative was to advance the use of data to improve decision-making, we developed standard metrics, established data collection systems, and built local capacity to incorporate metrics into routine practice. Although some cities had pre-existing digital surveillance systems in primary health settings, patient registries, or population-survey data, these systems were often not optimal. They were, for example, not integrated, fragmented across providers, or missing outcome indicators. At the start of the initiative, health output and outcome data were mainly stored in different registries as hard copies or not collected at all. As a result, data driven decision making required a true mindset shift for health authorities, managers, and providers alike. By for example translating progress and outcomes data into building useable dashboards for the health system managers and the city authorities, real-time data allowed the partnership to discuss, select, and readjust interventions as needed, and focus on areas where cascade data highlighted issues (e.g., early detection versus long term patient retention).

This initiative assessed the effect of hypertension control in public primary health centers. Given the co-existence of public and private healthcare provision in the cities, we acknowledge that this report may be biased by the exclusion of patients managed in the private sector. In the case of São Paulo, additional bias may be introduced through the analysis of only a sample of clinics, which were chosen on advice by the City Hall. While this directed sample aimed to cover the different models of care provision of the city, it is possible that the chosen care centers were not representative. Additionally, we are unable to assess the specific impact of interventions tackling underlying determinants of hypertension (e.g. reducing salt in processed food and introducing tobacco taxes in Ulaanbaatar, incentivizing workplace health and increasing access to healthy food options in Dakar or increasing access to physical exercise and health education in São Paulo). As CARDIO4Cities was integrated into the existing health system and ongoing hypertension management processes to put into practice existing international guidelines and best practices, and due to requirements set by the collaborating health authorities, a strict experimental setup (e.g. as a randomized control trial) was not possible. Additionally, the data collection systems to measure the initiative’s impact were set up as part of the initiative’s activities. Consequently, we were unable to establish a strict baseline or a no-intervention control group as comparator and the data presented in this manuscript should be interpreted as a description of the real-time progress made during implementation of CARDIO4Cities. We acknowledge that future replications of the CARDIO4Cities approach should investigate opportunities for implementation in a stricter experimental setup, to strengthen the quantitative evidence. Further, while all data was extracted from medical records and best practices in measurements and patient evaluation were promoted as part of this initiative, we were not involved in the generation of the data. For example, BP measurement followed local guidelines and may have been performed by nurses or physicians before the values were entered into the medical record, possibly by different individuals or at different time points. These differences in local practices may have affected data quality or introduced bias. Limitations in data quality may also have resulted from human errors in the manual data extraction from paper-based records, despite the standard data collection form developed by local partners. Data quality improved and missingness of data reduced over time. On the other hand, given that baseline data were collected while activities were already underway, we may underestimate the improvements by CARDIO4Cities. Further we observed ranges in mean systolic BP reduction but suppose that in the management of hypertension small changes in mmHg translate in a reduction of strokes [36]. Finally, this discussion provides additional context and possible explanations for the observed results. Our hypotheses are based in part on conversations with local partners and on impressions during implementation.

Currently, most evidence on improving BP control stems from high-income countries, reflecting continuous sustained efforts over prolonged periods [37, 38]. The short period of CARDIO4Cities implementation was intended to rapidly strengthen local capacity for hypertension management and integrate the model within primary health services. Our results indicate that comprehensive and coordinated multisector action is feasible, can improve hypertension control, and can rapidly be scaled in cities.

Overall, the CARDIO4Cities approach delivers first evidence to be an efficient solution to address CV risk factors such as hypertension and help narrow the health equity gap between high and low-resource populations within cities. Especially in urban settings with insufficient specialized health infrastructure to manage acute complications of hypertension, a simple yet efficient approach such as CARDIO4Cities can help avoid the devastating consequences of uncontrolled BP. This approach can be extended to address other CV risk factors and its replication could support many governments around the world to design and implement urgently needed roadmaps to improve urban CV population health.

## Supplementary Information

**Additional file 1: S1 Table.** Urban population health initiative’s implementation timeline and coverage. **S2 Table.** Urban population health initiative’s clinical data approach. **S3 Table.** Urban population health initiative Metrics. **S4 Table.** Examples of core interventions implemented in each city mapped on the CARDIO4Cities pillars. **S5 Table.** Urban population health initiative Implementation outputs (2017 - December 2019).

**Additional file 2. **Global Evaluation Requirements.

## Data Availability

The datasets generated and/or analyzed during the current study are not publicly available due to local approvals and ethical committees. Data was collected from standard procedures in the primary healthcare system and is thus under the jurisdiction of the respective health authorities. For Ulaanbaatar, data resides with Capital City Department of Health for Dakar with the Ministère de la Santé et de l’Action Sociale and for São Paulo with the Secretaria Municipal da Saúde in São Paulo, Brazil. The corresponding ethics approvals are Letter No. 1/158 dated February 21st, 2018 from the Capital City department of health for Ulaanbaatar, SEN 18/79 and SEN19/14 from the Comité National d’Ethique pour la Recherche en Santé for Dakar, and CEP-SMS; 3·818·858 for São Paulo. Insights into consolidated, aggregated data is available from the corresponding author on reasonable request. The indicator framework is provided in the supplementary material.

## Abbreviations

BP: Blood pressure
CI: Confidence intervals
CVD: Cardiovascular disease
DPRS: Direction de la Planification de la Recherche et de la Statistique
DSIS: Division de la Statistique et de l’Information Sanitaire
LMICs: Low- and middle-income countries
mmHg: Millimeter mercury
NA: Not applicable
OR: Odds ratio
Q: Quarter
REF: Reference
CEP-SMS: Comitê de Ética – Secretaria Municipal da Saúde, São Paulo
WHO: World Health Organization
