# Supplementary material for: Implementing a multisector public-private partnership to improve urban hypertension management in low-and middle- income countries
Source: BMC Public Health. 2022 Dec 19;22:2379. doi: 10.1186/s12889-022-14833-y (PMC9761621; doi:10.1186/s12889-022-14833-y)
Supplement: Supplementary file 1 — Additional file 1: S1 Table. Urban population health initiative’s implementation timeline and coverage. S2 Table. Urban population health initiative’s clinical data approach. S3 Table. Urban population health initiative Metrics. S4 Table. Examples of core interventions implemented in each city mapped on the CARDIO4Cities pillars. S5 Table. Urban population health initiative Implementation outputs (2017 - December 2019). [file 12889_2022_14833_MOESM1_ESM.docx]

**Supplement**

**S1 Table.** Urban population health initiative’s implementation timeline and coverage

|  | **Ulaanbaatar** | **Dakar** | **São Paulo** |
| --- | --- | --- | --- |
| **Program Installation Start^1^** | Sept 2017 | July 2017 | Dec 2017 |
| **Initial Implementation Evaluation Time Period, by district** | Feb–Jun 2018  Nov 2018–Sep 2019 | West: Apr 2018–Dec 2019  Center: July 2018–Dec 2019  North: July 2018–Dec 2019  South: Jan 2019–Dec 2019 | Oct 2018–Dec 2019 |
| **Participating Health Services Sites (% Primary Care Facilities in Area)** | 142 FHCs (100%) | 66 health centers, health posts, and community centers (100%):  West: 14  Center:13  North: 16  South: 23 | Itaquera: 24 UBSs (100%)  Penha: 21 UBSs (100%) |
| **Health Services Catchment Area** | 1·2 million | 1·3 million | 1·0 million |

1= Bertram RM, Blase KA, Fixsen DL. Improving programs and outcomes: Implementation frameworks and organization change. *Res Soc Work Pract* 2015; **25**:477^–^87.

FHCs=family health centres. UBS=Unidade Básica de Saúde.

**S2 Table.** Urban population health initiative’s clinical data approach

|  | **Ulaanbaatar** | **Dakar** | **São Paulo** |
| --- | --- | --- | --- |
| **Patients enrolled** | Patients, aged ≥18 years, registered in 25 clinics through Feb 2018, with documented (previous or new) hypertension*, prospectively followed for clinic visits during the initiative’s time period; new patients meeting eligibility criteria were enrolled from Feb 2018 through Sept 2019. | Prospective only: Patients, aged ≥18 years, visiting health sites through Dec 2019, with documented (previous or new)* hypertension; to be included in the program database, a new patient medical record had to be opened. | Prospective: Patients, aged ≥18 years, visiting UBSs who provided consent, with documented (previous or new)* hypertension; new patients meeting eligibility criteria were enrolled from Oct 2018 through Dec 2019; for analysis, additional criteria applied: patients had to have at least one valid BP measurement during initiative’s time period. |
| ***Criteria for hypertension diagnosis** | Documented notation of hypertension as diagnosis; BP≥130 and/or 80 mmHg. | Documented notation of hypertension as diagnosis; BP≥140 and/or 90 mmHg. | ICD-10 for arterial hypertension, documented prescription of anti-hypertensive medication; BP≥140 and/or 90 mmHg. |
| **Cardiovascular risk assessment** | Where: Assessed by physicians and documented in medical charts  Algorithm used: The WHO-ISH risk chart for WPR B Region, as recommended in the Mongolian Hypertension guidelines; Five categories (<10%, 10% to <20%, 20% to <30%, 30% to <40%, >40%) | Where: Assessed by physicians and documented in medical charts  Algorithm used: Adapted from the 2013 Practice guidelines for the management of arterial hypertension of the ESH and the ESC, as recommended in the hypertension algorithms developed by the urban population health initiative’s partners; Four categories: No risk; Low: <15%, Moderate: 15% to <20%, High: 20-30%, Very high: >30%) | Where: Assessed by physicians and documented in medical charts  Algorithm used: From a simplified hypertension algorithm developed by urban population health initiative’s partners, based on the 7^th^ Brazilian Guideline of Arterial Hypertension; Four risk categories: No additional risk; Low, Intermediate, High. |
|  | Categories in medical charts: Five WHO-ISH risk CV risk percent categories | Categories in medical charts: Low, Moderate, High; no individual scores available. | Categories in medical charts: No additional risk, Low, Moderate, High; no individual scores available. |
|  | Reported aggregate data: Low (<10%), Moderate (10-20%); High (≥20%) | Reported aggregate data: Low, Moderate, High (includes Very high) | Reported aggregated data: Low, Moderate; High |
| **Clinical data analysis - source** | Patient medical records from random sample of 23 clinics; originally 25, two clinics merged, and one dropped from the study. | Patient medical records from 66 healthcare sites using a staged approach aligned to program implementation. | Patient medical records from 6 UBSs in Itaquera district. Clinics were selected, as per guidance from the city Secretary of Health, to represent the different primary care management models in São Paulo. |
| **Clinical data analysis - time period** | Feb–Jun 2018  Oct–Sep 2019  Data collection interrupted in Q3 2018 following challenges with the timely development and widespread adoption of an online registry platform. For subsequent data collection, and to promote systematic and routine surveillance of hypertension management in FHCs, a simplified version of the original hypertension patient registration form was developed. | West: Apr 2018–Dec 2019  Center: July 2018–Dec 2019  North: July 2018–Dec 2019  South: Jan 2019–Dec 2019 | Prospective: Oct–Dec 2019  Retrospective: Oct 2017–Oct 2019 |
| **Clinical data collection process** | Each FHC designated one person trained as a data collector. Healthcare providers from each FHC used an Excel-based registry to gather data on patients with hypertension. The data collector gathered these data and submitted to the local M&E partner for analysis. | Eight data collectors, trained in the definition of indicators, use of a structured program-specific case report form, and analysis of data, who extracted data from paper-based patient records and clinic logbooks into electronic spreadsheets. | Patient consent was required prior to inclusion and access of medical records. Data collection performed by trained medical student interns from a local university using an Excel-based data collection tool. |

BP=blood pressure. CV=cardiovascular; ESC=European Society of Cardiology. ESC=European Society of Hypertension. FHC=family health center; ISH= International Society for Hypertension. M&E=measurement and evaluation. WHO=World Health Organization. WPR=Western Pacific Region.

*Previously diagnosed patients had a diagnosis of hypertension prior to their first visit during the urban population health initiative’s program period. In Dakar, previous diagnosis of hypertension was determined by a documentation of hypertension or antihypertensive medication in their patient record, or the documentation of the patient indicating to the healthcare provider that they were previously told to have hypertension or elevated blood pressure or that they had been prescribed antihypertensive medication. In São Paulo, previous diagnosis of hypertension was determined by notation of an ICD-10 code for hypertension, hypertension grade or hypertension medication in their patient file. In Ulaanbaatar, diagnosis of hypertension was determined by documentation of diagnosed hypertension in the patient records. Newly diagnosed patients had a first ever diagnosis hypertension during the urban population health initiative program period.

**S3 Table.** Urban population health initiative Metrics

|  | **Metric** | **Definition** |
| --- | --- | --- |
| **Diagnosis data, across all cities** | | |
| Within program | Cumulative number, Previous and new diagnosis | Cumulative total number of patients diagnosed with hypertension (refer to Tab S3 for criteria) |
| **Treatment data, across all cities** | | |
| Within program | Cumulative number | Cumulative total number of diagnosed patients prescribed medication treatment |
| Within program | Cumulative percent, within program | Cumulative total number of documented patients prescribed medication treatment / Cumulative total number of patients diagnosed with hypertension |
| **Control** |  |  |
| Within program | Cumulative percent, among diagnosed | Cumulative total number of patients with controlled BP at last known visit (assumes the patient has been diagnosed and has at least one follow-up visit, where BP was measured and documented) / Cumulative total number of patients diagnosed with hypertension |
| Within program | Cumulative percent, among treated | Cumulative total number of patients prescribed medications and have controlled BP at last known visit (assumes the patient has been diagnosed and has at least one follow-up visit, where BP was measured and documented) / Cumulative total number of documented patients prescribed medication treatment |
| **Shift in Mean SBP** | | |
| Within program | Absolute net change | Average of the difference in the SBP value between the first to last visit for all patients diagnosed with hypertension. Where a BP value may not have been recorded during the baseline visit, the BP measurement from the next available visit was used. |

BP=blood pressure. SBP=systolic blood pressure.

**S4 Table.** Examples of core interventions implemented in each city mapped on the CARDIO4Cities pillars.

The CARDIO4Cities pillars were implemented in the Better Hearts Better Cities initiative. The name of the initiative was localized to each geography when appliable (e.g. Better Hearts Better Ulaanbaatar – Ulaanbaatar; Better Hearts Better Cities – Dakar; Cuidando do Seu Coração – São Paulo).

**Ulaanbaatar**

| **Quality of Care** | **Ensure Access** | **Policy Reform** | **Data and digital** | **Intersectoral partnership** | **Local Ownership** |
| --- | --- | --- | --- | --- | --- |
| **Up dated and standardized hypertension guidelines** and their translation into simplified algorithms.  **Continued medical education for multi-disciplinary team** (HCP, nurses, clinic managers, social workers etc.) on standardized management of high BP. | **Proactive hypertension screening** and focus on **early detection** in primary health centres.  New component on **engagement of pharmacists in hypertension detection** and care with training and tools for adequate measurement and health information delivery. | Advocated for **health policy reform** e.g. primary care budget, dietary improvement strategies, tobacco taxes, etc.  Introduce **hypertension cascade indicators** and tracking of patient numbers in primary care.  **Cardiovascular risk scores** were routinely calculated and included into the evaluation of hypertensive patients. | Inform enhanced **data-collection mechanisms** for hypertension carried forward by the Ministry of Health and National Health Insurance. | **Health workplace initiatives** and introduced **physical and nutritional education in** schools, supporting to develop a curriculum for schools on information on NCD prevention.  Develop **early detection model** with community partners and implementing referral system.  Structure patient support and advocacy groups. | The Ministry of Health involvement in establishing **steering committees** focused on broad strategic direction, which included the city mayor and senior public managers with responsibilities for relevant sectors, including health, education, IT and communication, and agriculture. |

HCP: Health care professional; BP: blood pressure; NCD: non-communicable diseases; IT: information technology

**Dakar**

| **Quality of Care** | **Ensure Access** | **Policy Reform** | **Data and digital** | **Intersectoral partnership** | **Local Ownership** |
| --- | --- | --- | --- | --- | --- |
| **Standardized hypertension management** with clinical decision support for health providers and trainings.  Strengthen hypertension **referral systems to secondary and tertiary healthcare levels**. | **Systematic screening** of all adults >18 presenting in the city’s primary health centres.  **Community health awareness campaigns** with community activities.  **Introduction of task-shifting** to increase overall coverage and efficiency of primary health services. | Reinforce the routine calculation of **cardiovascular risk scores.** | **Enhanced hypertension data collection.** First consolidated paper-based hypertension registry to collect and monitor all hypertension data on district level in primary care. | Establish public private partnerships on **workplace and school programs** in revising the national curriculum for primary and secondary schools to integrate information on key CVD and NCD risk factors.  **Agricultural sector.** Public-private partners from the agricultural sector collaborated to increase the availability of fresh foods in the city.  Include medical societies to strengthen decision-making bodies around hypertension and NCDs management **policy decision-making** | **Ministry of Health leadership** through a multisector committee to address NCD and the joint development of the national strategy to address NCDs. |

CVD: cardiovascular disease; NCD: non-communicable diseases

**São Paulo**

| **Quality of Care** | **Ensure Access** | **Policy Reform** | **Data and digital** | **Intersectoral partnership** | **Local Ownership** |
| --- | --- | --- | --- | --- | --- |
| Develop a new, **standardized NCD management protocol** jointly with the City Hall, incl. algorithm of prevention and care  Capacity building for clinic managers through **management trainings and performance management system**  **Training of health providers and multi-disciplinary teams** on hypertension and CVD management through online continuous medical education.  Support strengthening the **role of pharmacists** in supporting treatment and treatment adherence. | Active screening and acceleration of early detection through the **implementation of BP self-screening corners** (in-clinic).  **Distribution of information material** promoting BP screening in all clinics in the province of Itaquera.  **Community engagement and mobilization** for opportunistic and out-of-office screening. | Introduce **targets for chronic disease management** in primary care centers and district supervision teams.  Emphasize **cardiovascular risk scores** and **data quality.** | **Strengthen hypertension** outcome and output **data collection** and include in monitoring activities.  Reinforce and train **primary care management with data**.  Introduce solutions to monitor **district coverage and targets**, **track numbers of hypertensive** patients. | **School-based program** aimed at raising **awareness on NCDs** (CEU – Amigo do Coração), incl. training of students and professional educational leaders on NCD management, risk factors, nutrition, physical education, and heart health literacy.  Build **partnerships with community champions** (e.g. football and samba clubs) to bring awareness and opportunistic screening possibilities. | **Leadership through the City Health Authorities** to establish coordinated care for chronic patients.  Establish **co-creation process in the design, optimization, and scale** of interventions. |

NCD: non-communicable diseases; CVD: cardiovascular disease; BP: blood pressure

**S5 Table.** Urban population health initiative Implementation outputs (2017 - December 2019)

| **Indicators** | **Ulaanbaatar** | **Dakar** | **São Paulo** | **Total** |
| --- | --- | --- | --- | --- |
| **Care** | | | | |
| Number of urban population health initiative clinics applying updated treatment protocols/care algorithms | 142 | 66 | 24 | 232 |
| **Training of clinic team** | | | | |
| Number of district health leadership members | 18 | 24 | 1 | 43 |
| Number of heads of clinics | 142 | 92 | 24 | 258 |
| Number of primary care doctors | 550 | 175 | 293 | 1,018 |
| Number of nurses, nursing assistants and midwives | 500 | 468 | 530 | 1,498 |
| Number of community health workers | 350 | 216 | 273 | 839 |
| Number of pharmacists | 500 | NA | 80 | 580 |
| Number of social workers | 117 | NA | NA | 117 |
| **Access** | | | | |
| Number of community health events held | 2177 | 168 | 1201 | 3546 |
| Number of people who attended community events | 60000 | 22196 | 5324 | 87520 |
| **Strengthened data collection system (Care, Access)** | | | | |
| Number of hypertension indicators incorporated into a mainstream or national surveillance system | 4 | 3 | 2 | NA |

NA=not applicable.
